# Neuropharmacological, Analgesic, and Anti‐Inflammatory Activities of *Pothos scandens* Acetone Extract With GC‐MS‐Based Phytochemical Profiling

**DOI:** 10.1155/bri/2685178

**Published:** 2026-04-22

**Authors:** S. M. Moazzem Hossen, Jannatul Naima Meem, Tanjina Nasrin Tamin, Rimon Chowdhury, Sadia Hosna Rony, Sheikh Imrul Kayes, Tawhidul Islam, Md. Liakot Ali

**Affiliations:** ^1^ Department of Pharmacy, Faculty of Biological Sciences, University of Chittagong, Chattogram, 4331, Bangladesh, cu.ac.bd; ^2^ Department of Pharmacy, BGC Trust University Bangladesh, Chattogram, 4381, Bangladesh, bgctub-edu.com; ^3^ Department of Applied Chemistry and Chemical Engineering, Faculty of Sciences, University of Chittagong, Chattogram, 4331, Bangladesh, cu.ac.bd; ^4^ Department of Pharmacy, International Islamic University Chittagong, Chattogram, 4318, Bangladesh, iiuc.ac.bd

**Keywords:** analgesic, anti-inflammatory, GC-MS, molecular docking, neuropharmacology, *Pothos scandens*

## Abstract

*Pothos scandens*, belonging to the Araceae family, locally known as “batilata,” is traditionally used for treating various chronic diseases in folk medicine, such as epilepsy, seizures, convulsions, wounds, snakebites, skin issues, asthma, muscle problems, and diarrhea. This research examined the anxiolytic, antidepressant, analgesic, and anti‐inflammatory potentials of the acetone extract of *P. scandens* whole plant (APS) on in vivo mice and rat models. The elevated plus maze (EPM) and hole board tests (HBTs) were carried out to assess the anxiolytic effects, while antidepressant activity with forced swimming test (FST) and tail suspension test (TST) and the acetic acid–induced writhing and formalin‐induced licking tests on Swiss albino mice were used to evaluate the analgesic activity, and the carrageenan‐induced rat paw edema model was applied for anti‐inflammatory effect. APS at 400 mg/kg exhibited significant anxiolytic (*p* < 0.001) and antidepressant (*p* < 0.01) effects. This study also demonstrated that APS has dose‐dependent and significant analgesic effects for acetic acid–induced writhing (*p* < 0.05) and formalin‐induced licking tests (*p* < 0.01), respectively, as well as anti‐inflammatory (*p* < 0.001) activities compared to the standard diclofenac sodium. Furthermore, 11 compounds were detected in the extract through GC‐MS. These phytochemicals exhibited significant binding affinities, excellent pharmacokinetics, and a safe toxicological profile, as reported by in silico molecular docking and ADMET analysis. The outcomes of this study confirmed that the presence of bioactive compounds in APS offers it considerable potential as a multimodal therapeutic substance with neuropharmacological, analgesic, and anti‐inflammatory properties.

## 1. Introduction

Anxiety and depression are among the most prevalent heterogeneous psychiatric disorders globally, significantly impacting mental health and overall well‐being. Neurological dysfunctions often result in fear responses that severely impair daily functioning and quality of life [1]. Nowadays, psychiatric conditions like anxiety and depression are among the most widespread health issues globally. These mental health disorders can vary in severity, from mild to severe. Anxiety disorders typically involve persistent fear or worry, often triggered by stressful or unfamiliar situations. Depressive disorders, on the other hand, may include symptoms such as persistent sadness, lack of interest, sleep disturbances, reduced appetite, difficulty carrying out everyday activities, and, in more serious cases, suicidal thoughts or behavior [2]. Imbalances in brain chemicals called monoamine neurotransmitters—specifically norepinephrine, serotonin, and dopamine—are believed to play a key role in causing depression [3].

Pain or algesia is an unpleasant sensation typically triggered by severe or harmful tissue damage. While its main role is to serve as a protective mechanism, it can sometimes become more detrimental than beneficial. To ease this discomfort, people use analgesics. These medications relieve pain by acting on the peripheral or central nervous systems [4]. Inflammation is the body’s natural physiological reaction to injury, infection, or harmful agents. During this process, hydroxyl radicals are produced in the affected tissues, which contribute to the increased expression of proinflammatory substances such as prostaglandins, inflammatory cytokines (like IL‐1β and IL‐16), and chemokines such as GRO‐alpha [5]. Pain is often associated with inflammation as a secondary effect caused by the release of certain chemical mediators. People worldwide have long relied on treatments like nonsteroidal anti‐inflammatory drugs (NSAIDs), corticosteroids, and immunosuppressants to manage inflammatory conditions. However, these medications can lead to undesirable side effects, including gastrointestinal bleeding and the development of peptic ulcers [6].

The utilization of natural products with therapeutic potential is a practice rooted in ancient human history. Since early times, plants, minerals, and animal‐derived substances—such as Epibatidine from the African clawed frog—have served as primary sources for drug development [7]. Plants possess a vast array of chemically diverse bioactive compounds, which makes them an important foundation for drug discovery research. For decades, medicinal agents derived from plants have been widely used in healthcare systems around the world [8, 9]. Throughout the progression of human civilization, plant‐derived natural compounds have not only been essential for the treatment of injuries and various diseases but have also carried cultural and ritualistic significance [10].

*Pothos scandens,* a member of the genus *Pothos* from the Araceae family, is an epiphytic plant that grows by climbing and produces roots along its branches. Its leaves grow in two rows and are shaped between oval and lance‐like, with pointed tips and a slightly slanted base. The leaf stalks are broad, flat, and end in a blunt shape, with a sheath at the base. The leaves are a vibrant green in color [11]. In the hilly areas of Bangladesh, indigenous communities refer to it as “Batilata” and have long utilized it in traditional medicine. They rely on the whole plant to help treat a variety of health problems, such as skin issues, asthma, snake bites, diarrhea, cancer, smallpox, sprains, epilepsy, convulsions, and wounds [12]. The crushed root, when fried in oil, is traditionally applied to treat abscesses. A leaf infusion is used as a remedy for seizures and epilepsy. Additionally, the stem, when combined with camphor and smoked like tobacco, is believed to help relieve symptoms of asthma [13].

Although *P. scandens* has been reported in traditional medicine and limited phytochemical investigations have identified several bioactive constituents, a comprehensive pharmacological evaluation of its neurobehavioral, analgesic, and anti‐inflammatory activities has not yet been systematically performed. In particular, no previous study has simultaneously investigated its anxiolytic and antidepressant effects using validated behavioral models, nor correlated its in vivo pharmacological actions with receptor‐level interactions. Therefore, the present study aimed to provide the first integrated assessment of the acetone extract of *P. scandens* using a combination of behavioral assays (EPM, HBT, FST, TST), nociceptive and inflammatory models, GC–MS‐based phytochemical profiling, and molecular docking analysis against relevant CNS and inflammatory targets (GABAA receptor, serotonin 5‐HT2A receptor, COX‐1, and COX‐2). This integrative approach is intended to bridge the gap between traditional use and mechanistic understanding, thereby providing scientific evidence supporting the therapeutic potential of this species.

## 2. Methods

### 2.1. Chemicals and Reagents

All experiments were carried out using high‐purity reagents procured from trusted suppliers. Acetone, acetic acid, and carrageenan were obtained from Sigma‐Aldrich Ltd. (St. Louis, USA). The reference drugs applied in the in vivo experiments—diclofenac sodium, diazepam, and fluoxetine—were sourced from Square Pharmaceuticals Ltd., Bangladesh.

### 2.2. Procurement of Plant Specimens and Extract Preparation

The entire *P. scandens* plant was collected in December 2024 from the campus of BGC Trust University, Bangladesh, situated in the Chittagong district. The species was taxonomically identified by Professor Dr. Shaikh Bokhtear Uddin of the Department of Botany, University of Chittagong, and recorded under voucher number CU/Pharm 1215. Following collection, the plant materials were cleaned, sun‐dried, and ground into a fine powder. Around 1300 g of this powder was macerated in 7 L of acetone at room temperature for 15 days. Cotton and Whatman No. 1 filter paper were used in succession to filter the resultant mixture. After that, the filtrate was concentrated at a specified temperature using a rotary evaporator with lowered pressure. After filtration and solvent evaporation, 29 g of crude extract was obtained, corresponding to a 2.23% (w/w) extraction yield. The extract was kept in a refrigerator for further use.

### 2.3. Gas Chromatography–Mass Spectrometry (GC–MS) Analysis

The phytochemical composition of APS was determined using GC–MS analysis, which was performed using a PerkinElmer GC–MS system (GC–Clarus 690 coupled with MS–SQ8C) equipped with an Elite‐5MS capillary column (30‐m × 0.25‐mm internal diameter × 0.25‐μm film thickness; Cat. No. N9316282). The stationary phase of the column consisted of 5% phenyl and 95% dimethylpolysiloxane. The oven temperature program was set as follows: the initial column temperature was maintained at 120°C for 2.0 min, then increased to 320°C at a ramp rate of 10°C/min, and finally held at 320°C for 10 min. The injector temperature was maintained at 220°C, while the interface and ion source temperatures were set at 200°C and 220°C, respectively. Helium was used as the carrier gas at a constant flow rate of 1.0 mL/min. Mass spectrometric detection was carried out in electron ionization (EI) mode with an ionization energy of 70 eV. Data were acquired in full scan mode over a mass range of m/z 50–450 amu, with a solvent delay of 2.0 min. Compound identification was achieved by comparing the obtained mass spectra with those in the NIST mass spectral library, and only compounds with a library match score ≥ 80% were considered tentatively identified.

### 2.4. Experimental Animals and Ethical Approval

Male Swiss albino mice (5–6 weeks old, 20–30‐g body weight) and male Wistar rats (5–6 weeks old, 100–120‐g body weight) were procured from the Animal House facility at the Department of Pharmacy, University of Chittagong. Before experimentation, all animals underwent a 7‐day acclimatization period under standardized laboratory conditions (25 ± 2°C, 55%–65% relative humidity) with a 12‐h light/dark photoperiod. Standard laboratory diet and purified water were provided to all animals throughout this period without restriction. The experimental protocol received formal ethical clearance (Approval No: AERB‐FBS‐CU‐2025109) from the Institutional Animal Ethics Review Board of the Faculty of Biological Sciences, University of Chittagong, Bangladesh.

### 2.5. Experimental Design

All in vivo experiments were performed on Swiss albino mice, except for the carrageenan‐induced paw edema model, which was conducted using Wistar rats. For each test except the anti‐inflammatory test, animals were randomly assigned to four groups (*n* = 5 per group) for pharmacological evaluation. Group 1 acted as the control and received only the vehicle (1% Tween‐80 in distilled water) 10 mg/kg (b.w., p.o.). Group 2 was administered a standard drug relevant to each specific test at specific dose. Groups 3 and 4 received APS at doses of 200 mg/kg and 400 mg/kg, b.w., p.o, respectively. All the reference drugs and extract dosages were dissolved in the vehicle solution. Diclofenac sodium was used as the reference drug for analgesic and anti‐inflammatory assessments, diazepam served as the standard for anxiolytic and sedative tests, and fluoxetine was used as the reference antidepressant.

### 2.6. In Vivo Anxiolytic Evaluation

#### 2.6.1. Elevated Plus Maze (EPM) Test

The EPM consisted of two open and two closed wooden arms (50 × 10 × 40 cm^3^), raised 40 cm above the floor. The “2.5. Experimental Design” section was followed while grouping and treating the mice. Each mouse was positioned in the middle of the maze, facing a closed arm, 60 minutes after treatment. The quantity of entries and the time spent in each arm were noted over 5 min. The experiment was conducted in a soundproof room [14].

#### 2.6.2. Hole Board Test (HBT)

The HBT used a wooden chamber (40 × 40 × 25 cm) with 16 evenly spaced holes (3 cm in diameter), elevated 25 cm above ground level. Following the same treatment protocol, mice were observed 1 hour after administration. For 5 min, the frequency of head dips was noted for each mouse [15].

### 2.7. In Vivo Antidepressant Evaluation

#### 2.7.1. Forced Swimming Test (FST)

A transparent cylindrical container (15 × 25 cm) filled with water maintained at 25 ± 2°C to a depth of 10 cm was used for the FST. Mice, treated as outlined in the “2.5. Experimental Design” section, were placed individually in the water 60 min post‐treatment. The test lasted 6 minutes: the first 2 minutes served as an adaptation phase, while immobility time was measured during the final 4 minutes [16].

#### 2.7.2. Tail Suspension Test (TST)

Using a similar treatment protocol with minor adjustments, mice were individually suspended by their tails. An adhesive bandage was applied roughly 1 cm from the tail tip, with the animals positioned 50 cm above the testing surface. Behavioral assessment was conducted over a 6‐min observation period, during which immobility time was quantified as periods without voluntary movement, excluding only essential respiratory motions [16].

### 2.8. In Vivo Analgesic Potential

#### 2.8.1. Acetic Acid–Induced Writhing Method

Pain was induced by intraperitoneal (i.p.) injection of 0.7% glacial acetic acid at a dose of 10 mL/kg. Mice were grouped and treated according to the “2.5. Experimental Design” section. In the standard group, treatment was administered 15 min before the acetic acid injection, whereas in the test groups, it was given 30 min prior. Five minutes after the acid was administered, the number of abdominal writhes was recorded over 20 min [17].

#### 2.8.2. Formalin‐Induced Paw Licking Method

For the formalin‐induced pain model, 20 μL of 1% formalin was injected subcutaneously into the right hind paw 60 min after treatment with APS and diclofenac sodium. Mice were grouped and treated as detailed in the “2.5. Experimental Design” section. Pain response was observed by recording the time (in seconds) spent licking or biting the injected paw during the early neurogenic phase (0–5 min) and the late inflammatory phase (15–30 min) postinjection [18]. The pain inhibition percentage was estimated using the appropriate formula as follows:

(1) % of pain inhibition=difference between reaction time of control group and treatment groupreaction time of control group×100.

### 2.9. In Vivo Anti‐Inflammatory Evaluation

Anti‐inflammatory activity was evaluated using the carrageenan‐induced paw edema model in male Wistar rats. Animals were randomly divided into five groups (*n* = 5 per group): (i) normal control (vehicle only, no carrageenan), (ii) negative control (vehicle + carrageenan), (iii) standard (diclofenac sodium + carrageenan), and (iv–v) test groups receiving the extract at 200 and 400 mg/kg (p.o.), respectively, followed by carrageenan. Acute inflammation was induced by subplantar injection of 100 μL of 1% carrageenan (w/v in 0.9% normal saline) into the right hind paw of all groups except the normal control. One hour before carrageenan administration, rats were treated orally with vehicle, diclofenac sodium (10 mg/kg), or the extract (200 or 400 mg/kg). Paw thickness was recorded using a slide/digital caliper immediately before carrageenan injection (0 h) and at 1, 2, 3, and 4 h post injection. The change in paw size relative to baseline was considered an index of edema [19].

### 2.10. In Silico Investigation

#### 2.10.1. Molecular Docking Investigation

GC–MS analysis of APS revealed 11 small molecules, which were downloaded from PubChem in 3D SDF format. For those available only in 2D, 3D structures were generated using Open Babel [20]. Ligands were then energy‐minimized and converted to .pdbqt format via AutoDockTools (v1.5.6). To determine the possible analgesic, anti‐inflammatory, anxiolytic, and antidepressant properties, the following protein structures were retrieved from the RCSB Protein Data Bank: cyclooxygenase‐1 (PDB ID: 5WBE), cyclooxygenase‐2 (PDB ID: 5IKR), human GABA_A_ receptor alpha1‐beta2‐gamma2 subtype (PDB ID: 6X3W), and human serotonin 2A receptor (PDB ID: 7WC4). Protein preparation involved removing water and heteroatoms in Discovery Studio 2020 [21], followed by energy minimization using Swiss‐PDB Viewer [22]. AutoDockTools was subsequently utilized to convert the cleaned PDB files to PDBQT format. Molecular docking was executed employing PyRx AutoDock Vina [23, 24], where proteins were kept rigid and ligands flexible. Grid boxes were defined around the active sites of each target protein using AutoDock parameters. For all proteins, a uniform grid box size of 25 × 25 × 25 Å was applied. The grid box centers were set as follows: cyclooxygenase‐1 (PDB ID: 5WBE) at *X* = 36.65, *Y* = 162.85, *Z* = 24.82; cyclooxygenase‐2 (PDB ID: 5IKR) at *X* = 27.71, *Y* = 162.67, *Z* = 24.23; human GABA_A_ receptor alpha1‐beta2‐gamma2 subtype (PDB ID: 6X3W) at *X* = 109.71, *Y* = 94.09, *Z* = 106.08; and human serotonin 2A receptor (PDB ID: 7WC4) at *X* = −26.87, *Y* = −15.25, *Z* = 144.80. Docking calculations were performed with an exhaustiveness value of 8, and nine docking poses were generated for each protein. The top‐ranked (lowest binding energy) pose was selected for subsequent analysis. Docking interactions were visualized in both 2D and 3D using Discovery Studio 2020.

#### 2.10.2. ADMET Investigation

The pharmacokinetic (ADME) and toxicity profiles of the compounds were evaluated using two popular online tools, SwissADME [25] and pkCSM [26]. Additionally, compliance with Lipinski’s Rule of Five (Ro5) was examined to determine the compounds’ drug‐likeness.

### 2.11. Statistical Analysis

The results are presented as mean ± SEM. Statistical evaluation was conducted using one‐way ANOVA, with Dunnett’s *t*‐test applied for post hoc comparisons. Significance levels were defined as *p* < 0.001, *p* < 0.01, and *p* < 0.05 versus the control group. All analyses were performed using GraphPad Prism (version 5.2).

## 3. Result

### 3.1. GC–MS Profiling

**FIGURE 1:**
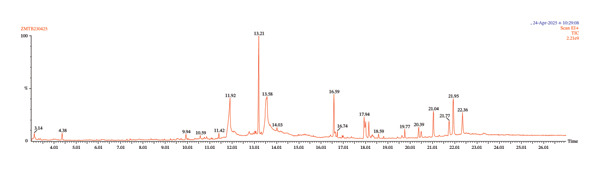
GC–MS chromatogram of acetone extract of *Pothos scandens* whole plant.

**FIGURE 2:**
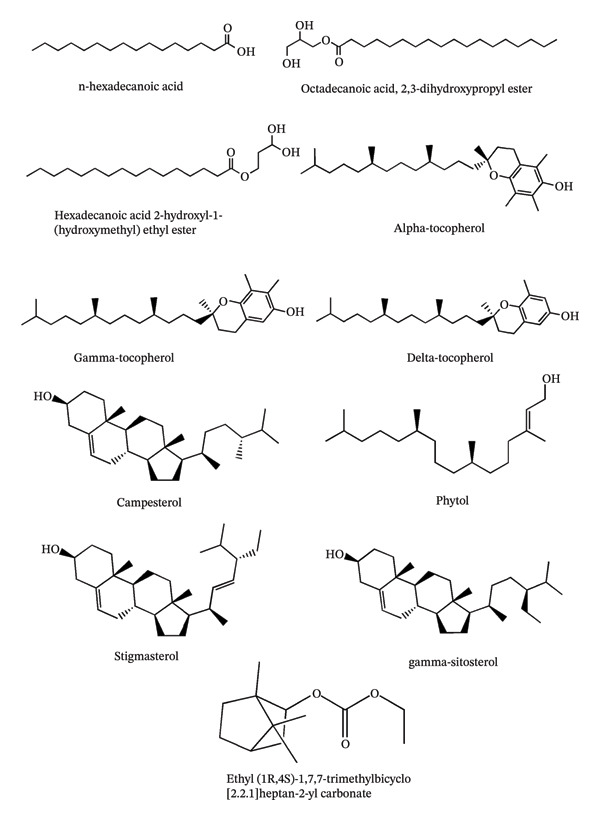
2D representation of the compounds identified from the acetone extract of the *Pothos scandens* whole plant.

The APS whole plant was subjected to GC–MS analysis, which revealed 11 distinct chemical compounds. The chromatogram and the 2D structures of these compounds are displayed in Figures 1 and 2, respectively. Relevant details on the detected chemical components are provided in Table 1.

**TABLE 1:** List of compounds identified in the acetone extract of the *Pothos scandens* whole plant by gas chromatography–mass spectrometry analysis.

SL.no	RT	Compound name	M.W.	Formula	Reverse match factor	Forward match factor	% of area
1	4.38	Ethyl (1R,4S)‐1,7,7‐trimethylbicyclo[2.2.1]heptan‐2‐yl carbonate	226	C_13_H_22_O_3_	911	862	1.34
2	11.92	n‐Hexadecanoic acid	256	C_16_H_32_O_2_	864	830	28.91
3	13.21	Phytol	296	C_20_H_40_O	917	901	19.54
4	16.59	Hexadecanoic acid 2‐hydroxy‐1‐(hydroxymethyl) ethyl ester	330	C_19_H_38_O_4_	893	833	13.42
5	18.15	Octadecanoic acid, 2,3‐dihydroxypropyl ester	358	C_21_H_42_O_4_	865	826	2.52
6	19.77	Delta‐tocopherol	402	C_27_H_46_O_2_	881	795	2.60
7	20.39	Gamma‐tocopherol	416	C_28_H_48_O_2_	891	785	2.30
8	21.04	Alpha‐tocopherol	430	C_29_H_50_O_2_	906	843	7.19
9	21.77	Campesterol	400	C_28_H_48_O	853	675	3.98
10	21.95	Stigmasterol	412	C_29_H_48_O	934	816	10.93
11	22.36	Gamma‐sitosterol	414	C_29_H_50_O	878	679	7.27

### 3.2. Anxiolytic Activity

#### 3.2.1. EPM Test

In the EPM test, the reference drug diazepam significantly (*p* < 0.001) prolonged the time stayed in the open arms, as seen in Table 2. However, mice given *P. scandens* extracts at 200 and 400 mg/kg showed a tendency toward more time stayed in these arms when contrasted with the control. The 400 mg/kg of APS led to a considerably (*p* < 0.001) longer duration stayed in the open arms. Besides, the entries in the open arms also increased significantly (*p* < 0.05, *p* < 0.01) in a dose‐dependent manner. Thus, the extract can be considered a potent anxiolytic at the high dose.

**TABLE 2:** Evaluation of the anxiolytic action of APS through the EPM test.

Treatment (mg/kg)	Time spent in open arms(s)	Entries in open arms	Time spent in close arms(s)	Entries in close arms
Control	180.4 ± 6.22	4.30 ± 0.20	119.6 ± 6.22	10.4 ± 0.93
Diazepam (1 mg/kg)	280 ± 4.72^∗∗∗^	10 ± 1.42^∗∗∗^	20 ± 4.72^∗∗∗^	8.20 ± 1.07
APS 200	193.8 ± 5.84	7.80 ± 0.58^∗^	106.2 ± 5.84	9.40 ± 0.75
APS 400	263.6 ± 4.78^∗∗∗^	9.20 ± 0.86^∗∗^	36.4 ± 4.78^∗∗∗^	7.00 ± 0.71^∗^

*Note:* Quantitative data are expressed as mean ± SEM (*n* = 5). Statistical analysis was performed using one‐way ANOVA with Dunnett’s post‐test, with significance thresholds set at  ^∗^ *p* < 0.05,  ^∗∗^ *p* < 0.01, and  ^∗∗∗^ *p* < 0.001 compared to control groups. APS represents the acetone extract of *Pothos scandens*.

#### 3.2.2. HBT

**FIGURE 3:**
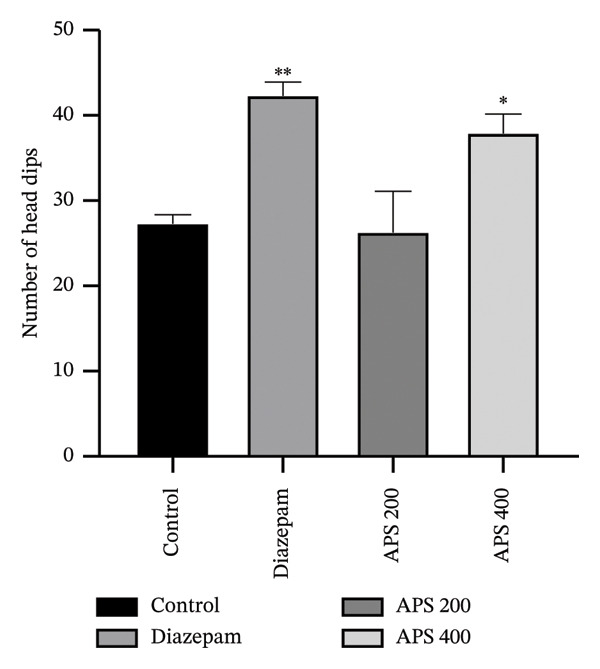
Effect of the acetone extract of *Pothos scandens* on the hole board test. Data were presented as mean ± SEM (*n* = 5), and  ^∗∗^ *p* < 0.01 and  ^∗^ *p* < 0.05 were considered significant. APS, acetone extract of *Pothos scandens*.

When comparing the diazepam‐treated mice to the positive control animals, the number of head dips was considerably higher (*p* < 0.01). When compared to the positive control mice, APS at a 400‐mg/kg dosage level considerably enhanced the number of head dipping (*p* < 0.05), which was similar to the EPM (Figure 3).

### 3.3. Antidepressant Activity

**FIGURE 4:**
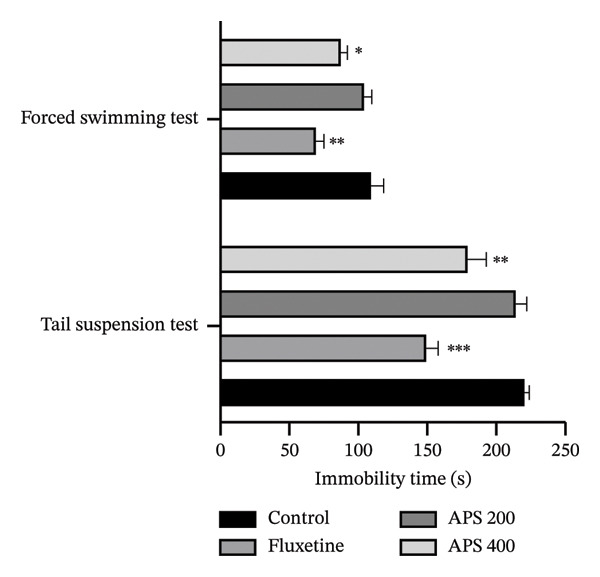
Antidepressant effect of the acetone extract of *P. scandens* on FST and TST. Data were presented as mean ± SEM (*n* = 5), and  ^∗∗∗^ *p* < 0.001,  ^∗∗^ *p* < 0.01, and  ^∗^ *p* < 0.05 were considered significant. APS, acetone extract of *Pothos scandens*.

As shown in Figure 4, APS administration significantly influenced immobility duration in both FST and TST. At doses of 200 and 400 mg/kg, APS induced a dose‐dependent diminution in immobility time relative to vehicle‐treated controls, with the higher dose (400 mg/kg) achieving statistical significance in both paradigms (*p* < 0.05 for FST and *p* < 0.01 for TST). The reference antidepressant fluoxetine (10 mg/kg) similarly reduced immobility duration (*p* < 0.01 in FST and *p* < 0.001 in TST). The 400‐mg/kg APS treatment demonstrated equivalent behavioral effects to fluoxetine administration in both behavioral models, supporting its potential as a comparable antidepressant agent.

### 3.4. Analgesic Activity

#### 3.4.1. Acetic Acid–Induced Writhing

**FIGURE 5:**
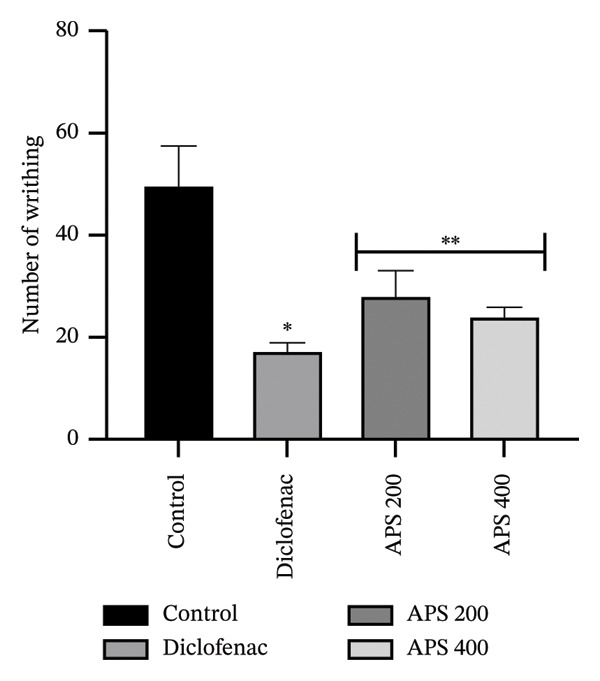
Analgesic effect of the acetone extract of *P. scandens* by acetic acid–induced writhing test. Data were presented as mean ± SEM (*n* = 5), and  ^∗∗∗^ *p* < 0.001 and  ^∗∗^ *p* < 0.01 were considered significant. APS, acetone extract of *Pothos scandens*.

In the acetic acid–induced writhing model, the reference medication diclofenac sodium (10 mg/kg) and the extracts (200 and 400 mg/kg) significantly decreased abdominal writhing (*p* < 0.001 and *p* < 0.01, respectively) compared to the control (Figure 5).

#### 3.4.2. Formalin‐Induced Paw Licking

In the formalin‐induced paw licking test, diclofenac sodium (10 mg/kg) produced a significant reduction in paw licking time in both the early (0–5 min) and late (15–30 min) phases, showing 23.55% and 50.62% inhibition, respectively, compared to the control group. APS at 200 mg/kg showed minimal inhibition in the early phase (3.61%) but markedly reduced paw licking time in the late phase (31.79%). Treatment with APS at 400 mg/kg produced greater inhibition in both phases, with 10.25% inhibition in the early phase and a significant reduction in the late phase (40.82%). These findings indicate a dose‐dependent analgesic effect of APS, predominantly during the inflammatory (late) phase of the formalin test (Table 3).

**TABLE 3:** Assessment of analgesic effect of acetone extract of *Pothos scandens* whole plant through formalin‐induced paw licking test.

Treatment (mg/kg)	0–5 min (early phase) paw licking time (sec)	Pain inhibition (%)	15–30 min (late phase) paw licking time (sec)	Pain inhibition (%)
Control	72.2 ± 1.77	—	177.4 ± 34.2	—
Diclofenac sodium (10 mg/kg)	55.2 ± 3.15^∗∗^	23.55	87.6 ± 2.46^∗^	50.62
APS 200	69.6 ± 2.42	3.61	121 ± 16.53	31.79
APS 400	64.8 ± 3.95	10.25	105 ± 24.47^∗^	40.82

*Note:* Quantitative data are expressed as mean ± SEM (*n* = 5). Statistical analysis was performed using one‐way ANOVA with Dunnett’s post‐test, with significance thresholds set at  ^∗^ *p* < 0.05,  ^∗∗^ *p* < 0.01, and  ^∗∗∗^ *p* < 0.001 compared to control groups. APS represents the acetone extract of *Pothos scandens*.

### 3.5. Anti‐Inflammatory Activity

#### 3.5.1. Carrageenan‐Induced Rat Paw Edema

In the carrageenan‐induced model, the standard treatment demonstrated notable reductions in paw circumference, achieving a 70.39% edema inhibition (*p* < 0.001^∗∗∗^) at 4 h post‐treatment. The APS showed a considerable dose‐dependent anti‐inflammatory effect at 200 and 400 mg/kg, and at the 4‐h mark, the higher dose produced a 55.86% inhibition (*p* < 0.001^∗∗∗^), as mentioned in Table 4.

**TABLE 4:** Anti‐inflammatory action of acetone extract of *Pothos scandens* whole plant on carrageenan‐induced rat paw edema.

Treatment (mg/kg)	Preinjection mean paw thickness (mm)	Postinjection mean paw thickness (mm) (% of edema inhibition)			
		1 h	2 h	3 h	4 h
Control	3.13 ± 0.049	5.31 ± 0 0.046	5.19 ± 0.036	5.12 ± 0.01	4.92 ± 0.207
		—	—	—	—

Diclofenac sodium (10 mg/kg)	3.12 ± 0.065	5.26 ± 0.043 1.84%	4.18 ± 0.074^∗∗∗^ 48.54%	3.92 ± 0.028^∗∗∗^ 59.79%	3.65 ± 0.027^∗∗∗^ 70.39%

APS 200	3.06 ± 0.01	5.11 ± 0.043^∗∗^ 5.96%	4.24 ± 0.018^∗∗∗^ 42.71%	4.20 ± 0.017^∗∗∗^ 42.72%	4.12 ± 0.015^∗∗∗^ 40.78%

APS 400	3.04 ± 0.011	5.05 ± 0.022^∗∗∗^ 7.79%	4.17 ± 0.065^∗∗∗^ 45.15%	3.92 ± 0.154^∗∗∗^ 55.78%	3.83 ± 0.146^∗∗∗^ 55.86%

*Note:* Quantitative data are expressed as mean ± SEM (*n* = 5). Statistical analysis was performed using one‐way ANOVA with Dunnett’s post‐test, with significance thresholds set at  ^∗^ *p* < 0.05,  ^∗∗^ *p* < 0.01, and  ^∗∗∗^ *p* < 0.001 compared to control groups. APS represents the acetone extract of *Pothos scandens*.

### 3.6. Molecular Docking Simulation Analysis

Computer‐aided drug research has provided a new and creative avenue to new drug discovery [27]. Molecular docking is frequently employed to anticipate ligand–protein interactions and to obtain more precise data on the biological activity of organic substances. It also gives details on how different proteins interact and potential modes of action of their binding sites [28]. Table 5 represents the docking scores of the compounds selected from the APS against the human GABA_A_ receptor alpha1‐beta2‐gamma2 subtype (PDB ID: 6 × 3W), serotonin 2A receptor (PDB ID: 7WC4), cyclooxygenase‐1 (PDB ID: 5WBE), and cyclooxygenase‐2 (PDB ID: 5IKR) for anxiolytic, antidepressant, anti‐inflammatory, and analgesic activities.

**TABLE 5:** Docking scores (kcal/mol) of the identified compounds from the APS extract against the human GABA_A_ receptor alpha1‐beta2‐gamma2 subtype (PDB ID: 6X3W), serotonin 2A receptor (PDB ID: 7WC4), cyclooxygenase‐1 (PDB ID: 5WBE), and cyclooxygenase‐2 (PDB ID: 5IKR) for anxiolytic, antidepressant, anti‐inflammatory, and analgesic potentials.

Phytochemicals	Pubchem ID	Receptors			
		Anxiolytic	Antidepressant	Analgesic and anti‐inflammatory	
		6X3W	7WC4	5WBE	5IKR
Ethyl (1R,4S)‐1,7,7 trimethylbicyclo[2.2.1] heptan‐2‐yl carbonate	23,035,345	−4.7	−6.9	−6.7	−4.9
n‐Hexadecanoic acid	985	−4.4	−7	−5.8	−6.4
Phytol	5,280,435	−5.3	−7.9	−6.4	−6.4
Hexadecanoic acid 2‐hydroxy‐1‐(hydroxymethyl) ethyl ester	129,853,056	−4.4	−6.8	−6.2	−6.4
Octadecanoic acid, 2,3‐dihydroxypropyl ester	24,699	−4.7	−7.1	−5.7	−6.6
Delta‐tocopherol	92,094	−6.1	−9.8	**−8.1**	**−6.7**
Gamma‐tocopherol	92,729	−6.4	−10.7	−8	−6.6
Alpha‐tocopherol	14,985	−6.1	**−10.8**	−8	−5.5
Campesterol	173,183	−5.8	−9.9	−7.1	−4.9
Stigmasterol	5,280,794	−6.9	−9.3	−6.6	−4.4
gamma‐Sitosterol	457,801	**−7**	−10.3	−5.6	−5.2
Standard (diazepam/fluoxetine/diclofenac)	3016/3386/3033	−6.8	−9.5	−7.3	−8.2

*Note:* Highest binding affinity is presented as bold.

#### 3.6.1. Docking Analysis for Anxiolytic Potential

**FIGURE 6:**
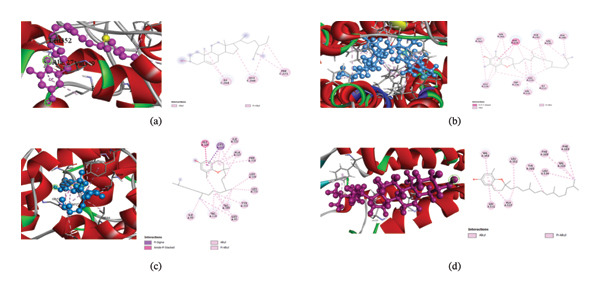
Molecular docking interactions of the top‐docked compound with each selected drug target protein. (a) Human GABA_A_ receptor alpha1‐beta2‐gamma2 subtype (PDB ID: 6X3W) vs gamma‐sitosterol. (b) Serotonin 2A receptor (PDB ID: 7WC4) vs alpha‐tocopherol. (c) Cyclooxygenase‐1 (PDB ID: 5WBE) vs delta‐tocopherol. (d) Cyclooxygenase‐2 (PDB ID: 5IKR) vs delta‐tocopherol.

In this research, the anxiolytic docking analysis was carried out using the human GABA_A_ receptor alpha1‐beta2‐gamma2 subtype (PDB ID: 6X3W) (Table 6). The APS extract’s compounds showed binding affinity scores against this protein that ranged from −7 to −4.4. With a score of −7 kcal/mol, the molecule gamma‐sitosterol showed the greatest binding affinities, surpassing that of the standard inhibitor diazepam (−6.8 kcal/mol). Six hydrophobic interactions with three active site amino acid residues Phe‐221, Ile‐264, and Leu‐268 interacted hydrophobically with this molecule (Figure 6(a)). According to the docking score, gamma‐sitosterol, stigmasterol, and gamma‐tocopherol are the top three compounds.

**TABLE 6:** Docking scores of the top‐docked compounds identified from *Pothos scandens* with the human GABA_A_ receptor alpha1‐beta2‐gamma2 subtype (PDB ID: 6X3W).

Compound name	Binding affinity (kcal/mol)	Hydrogen bonds		Hydrophobic bonds		
		Conventional	Carbon hydrogen	Pi‐alkyl	Alkyl	Others
Gamma‐sitosterol	−7			Phe‐221 (2)	Ile‐264 (2), Leu‐268 (2)	
Stigmasterol	−6.9				Pro‐228 (2), Leu‐231 (2), Ile‐232, Leu‐235, Ile‐264 (3), Leu‐268 (2)	
Gamma‐tocopherol	−6.4			Tyr‐220, Phe‐221	Leu‐223 (2), Pro‐184, Leu‐268	Pi‐Sigma: Tyr‐220
Diazepam (standard)	−6.8	ASN‐217		Tyr‐220, Leu‐223	Leu‐223	Pi‐Pi Stacked: Tyr‐220. Pi‐Pi T‐shaped: Tyr‐220. Amide‐Pi Stacked: Gly‐219, Tyr‐220

#### 3.6.2. Docking Analysis for Antidepressant Potential

Table 7 presents aspects of the docking analysis results on the antidepressant action. The human serotonin 2A receptor (PDB ID: 7WC4) was employed in this investigation to conduct screening tests for prospective antidepressant compounds. Compounds from the APS extract exhibited binding affinities against this protein that varied from −10.8 to −6.8. The top three drugs including alpha‐tocopherol, gamma‐tocopherol, and gamma‐sitosterol, all outperformed the common inhibitor fluoxetine (−9.5 kcal/mol) with binding affinities of −10.8, −10.7, and −10.3 kcal/mol, respectively. The top chemical compound, alpha‐tocopherol, established 23 hydrophobic interactions with 11 active site amino acid residues: Phe‐243, Trp‐336, Phe‐339, Phe‐340, Val‐156, Val‐366, Ile‐152, Leu‐228, Leu‐229, Leu‐362, and Val‐235 (Figure 6(b)). Alpha‐tocopherol, gamma‐tocopherol, and gamma‐sitosterol are the top three compounds based on docking score.

**TABLE 7:** Docking scores of the top‐docked compounds identified from *Pothos scandens* with the serotonin 2A receptor (PDB ID: 7WC4).

Compound name	Binding affinity (kcal/mol)	Hydrogen bonds		Hydrophobic bonds		
		Conventional	Carbon hydrogen	Pi‐alkyl	Alkyl	Others
Alpha‐tocopherol	−10.8			Phe‐243 (3), Trp‐336 (2), Phe‐339 (3), Phe‐340, Leu‐228, Leu‐362	Val‐156, Val‐366 (2), Ile‐152, Leu‐228 (2), Leu‐229 (3), Leu‐362, Val‐235	Pi–Pi T‐shaped: Phe‐339
						
Gamma‐tocopherol	−10.7		Leu‐229	Phe‐243 (3), Trp‐336 (2), Phe‐339 (4), Phe‐340, Leu‐228, Leu‐362	Val‐156, Val‐366, Leu‐229 (3), Leu‐228 (2), Leu‐362, Ile‐152, Val‐235	Pi‐Pi T‐shaped: Phe‐339
Gamma‐sitosterol	−10.3		Asn‐363	Trp‐151 (2), Phe‐243 (2), Phe‐339 (3), Phe‐340 (2)	Val‐156 (2), Val‐366, Ile‐152, Leu‐229 (2), Leu‐228 (2), Val‐235	Pi‐sigma: Phe‐243
						
Fluoxetine (standard)	−9.5	Val‐235, Ser‐239	Phe‐234	Phe‐243, Trp‐336 (2), Phe‐340, Leu‐229	Phe‐243, Trp‐336 (2), Phe‐340, Leu‐229	Halogen (Fluorine: Halogen Pi‐Pi Stacked: Phe‐243 Pi‐Pi T‐shaped: Phe‐339 (2)

#### 3.6.3. Docking Analysis for Analgesic and Anti‐Inflammatory Potential

The docking analysis outcomes for the analgesic and anti‐inflammatory actions of the extract components are shown in Tables 8 and 9. The cyclooxygenase‐1 (PDB ID: 5WBE) and cyclooxygenase‐2 (PDB ID: 5IKR) were used to check for possible analgesic and anti‐inflammatory substances. The compounds isolated from the acetone extract have binding affinities ranging from −8.1 to −5.6 kcal/mol and −6.7 to −4.4 kcal/mol against the cyclooxygenase‐1(PDB ID: 5WBE) and cyclooxygenase‐2 (PDB ID: 5IKR) receptors, respectively. The compound with the highest binding affinity among them was delta‐tocopherol.

**TABLE 8:** Docking scores of the top‐docked compounds identified from *Pothos scandens* with the cyclooxygenase‐1 (PDB ID: 5WBE).

Compound name	Binding affinity (kcal/mol)	Hydrogen bonds		Hydrophobic bonds	
		Conventional	Carbon hydrogen	Pi‐alkyl	Alkyl
Delta‐Tocopherol	−8.1			Tyr‐355, Ala‐527	Val‐116, Val‐119, Ala‐527, Val‐349, Leu‐352, Ile‐89
Alpha‐Tocopherol	−8.0	Arg‐120		Tyr‐355, Tyr‐385, Trp‐387, Phe‐518, Ile‐89	Val‐116, Ile‐523, Ala‐527 (2), Leu‐352
Gamma‐Tocopherol	−8.0‐			Tyr‐355, Leu‐352	Leu‐112, Leu‐115, Val‐116 (3), Ala‐527, Ala‐527, Val‐119, Arg‐120
Diclofenac (standard)	−7.3	Tyr‐355		Leu‐352, Ala‐527 (2), Val‐349	

**TABLE 9:** Docking scores of the top‐docked compounds identified from *Pothos scandens* with the cyclooxygenase‐2 (PDB ID: 5IKR).

Compound name	Binding affinity (kcal/mol)	Hydrogen bonds		Hydrophobic bonds	
		Conventional	Carbon hydrogen	Pi‐alkyl	Alkyl
Delta‐Tocopherol	−6.7			Phe‐205, Phe‐209, Tyr‐385, Val‐349, Ala‐527, Leu‐531	Leu‐352, Ala‐527, Val‐228, Leu‐534
Octadecanoi acid‐2,3 dihydroxy propyl ester	−6.6	Tyr‐355		Phe205 (2), Phe‐209, Phe381 (3), Tyr‐385 (3), Trp‐387, Phe‐518	Val‐349, Leu‐534, Met‐522, Leu‐352 (2)
Gamma‐Tocopherol	−6.6			Phe‐205, Phe‐209 (2), Phe‐381, Tyr‐385, Val‐349, Ala‐527, Leu‐531	Leu‐352, Val‐523, Ala‐527, Val‐228, Leu‐534
Diclofenac (standard)	−8.2	Tyr‐385		Ala‐527, Val‐349, Leu‐531	

### 3.7. ADMET Prediction

ADMET studies were carried out to ascertain the pharmacokinetic and toxicity parameters of the compounds detected in the APS from GC–MS analysis (Table 10). All the identified compounds met Lipinski’s rule of five, suggesting that they had desirable drug‐like attributes. It was determined that none of the compounds were AMES or hepatotoxic.

**TABLE 10:** ADMET and drug‐likeliness analysis of the compounds identified from the acetone extract of *Pothos scandens* whole plant.

Name of compound	Absorption		Distribution		Metabolism	Excretion	Toxicity
	Water solubility (log mol/L)	Intestinal absorption (human) (% absorbed)	VDss (human) (log L/kg)	BBB permeability (log BB)	CYP3A4 substrate	Total clearance (log ml/min/kg)	AMES toxicity
Ethyl (1R,4S)‐1,7,7‐trimethylbicyclo [2.2.1] heptan‐2‐yl carbonate	−3.303	93.893	0.292	0.732	No	0.935	No
n‐Hexadecanoic acid	−5.562	92.004	−0.543	−0.111	No	1.763	No
Phytol	−7.535	90.643	0.385	0.793	No	1.686	No
Hexadecanoic acid 2‐hydroxy‐1‐(hydroxymethyl) ethyl ester	−5.463	90.441	−0.321	−0.857	No	1.936	No
Octadecanoic acid, 2,3‐dihydroxypropyl ester	−6.044	90.234	−0.288	−0.911	No	2.04	No
Delta‐tocopherol	−7.812	89.6	0.902	0.698	No	0.824	No
Gamma‐tocopherol	−7.602	90.043	0.732	0.739	No	0.829	No
Alpha‐tocopherol	−6.901	89.782	0.709	0.876	No	0.794	No
Campesterol	−6.898	94.757	0.29	0.771	No	0.572	No
Stigmasterol	−6.682	94.97	0.178	0.771	No	0.618	No
Gamma‐sitosterol	−6.773	94.464	0.193	0.781	No	0.628	No

## 4. Discussion

The most prominent mood disorders are neurological diseases like anxiety and depression, which pose a persistent threat to public health. Acute and chronic pain have an intense reciprocal connection with anxiety and depression, making it one of the most likely triggers of these disorders, even if the exact cause of these conditions is still unresolved [29, 30]. It is interesting to note that the neural pathways, proinflammatory cytokines, neurotransmitters, and clinical manifestations of depression and pain appear comparably. Furthermore, the brain and nervous system processes regulating pain and depression signaling pathways are similar to neurotransmitters like serotonin and norepinephrine. As a result, those who have this kind of chronic pain may experience anxiety and emerging depression [31]. A longer duration of acute pain results in more mood dysregulation, whereas depression and anxiety are linked to a greater impact of pain intensity. While there is evidence linking both anxiety and depression to acute pain, the relationship between depression and acute pain has been investigated in greater detail [30].

Traditional medicine across the world relies on a variety of medicinal plants to treat depression, anxiety, and pain since ancient times [32, 33]. Meanwhile, the use of novel animal prototypes and rigorously validated testing is required to produce traditional lead molecules from medicinal plants with a variety of pharmacological effects, ensuring a trustworthy preclinical and clinical outcome [31]. As a result, our current research aims to investigate the neuropharmacological, analgesic, and anti‐inflammatory efficacies of APS using combined behavioral assays and molecular modeling studies.

The neurotransmitters, especially 5‐hydroxytryptamine (serotonin), gamma‐aminobutyric acid (GABA), and other amino acids and their metabolites, are implicated in the underlying mechanisms of anxiety and depression [34]. The EPM and HBT tests were utilized to validate our plant extract’s anxiolytic activity. A more accurate tool for evaluating anxious behavior is thought to be the EPM test. Accelerated entrances and duration in the open arms are manifestations of an anxiolytic potential in this test [35]. Our extract exhibited a significant (*p* < 0.001) anxiolytic action at the higher dose (400 mg/kg), with prolonged entrances and duration in the open arm. A similar result was seen in HBT. The higher frequency of head dips in HBT indicates suspicious behavior. In this case, diazepam, a highly potent anxiolytic, was employed as a reference standard [36]. The administration of diazepam and the APS extract at 400 mg/kg in our investigation entailed a moderately significant (*p* < 0.05) quantity of head‐dipping and a delay in the initial head‐dipping compared to the control. Numerous animal experiments indicate that GABAergic neurotransmission within the amygdala plays a crucial role in controlling anxiety‐related behaviors. The α‐subunits of the GABA_A_ receptor regulate the receptor’s responsiveness to GABA and largely define its pharmacological specificity toward drugs acting at the same binding site [37]. Our molecular docking results indicated that APS phytochemicals interacted favorably with the GABA_A_ receptor, showing higher binding affinity. Consequently, the anxiolytic effect of APS may partly arise from GABA_A_ receptor modulation along with other possible pathways. The antidepressant potential of the *P. scandens* extract was evaluated using the most common methods, FST and the TST. Animals in both models were placed in an inescapable movement. They became motionless, simulating a state of behavioral despair that resembles human depression [38]. Reduction in the immobility time of the animals during tests indicates that a sample has antidepressant action [39]. Our result indicated that APS 400 showed a notable antidepressant effect by reducing the immobility time in both FST (*p* < 0.05) and TST (*p* < 0.01) models. Serotonin 2A receptors occur across multiple brain regions, showing the highest abundance in the neocortex. Research has linked these receptors to the onset of depression and to the pharmacological effects of antidepressant medications [40]. The molecular docking results demonstrated potential binding interactions between APS constituents and the serotonin 2A receptor, indicating that this receptor might be involved in the antidepressant‐like activity of APS. Nevertheless, additional experimental validation is necessary to clarify the exact mechanism.

The analgesic activity of APS was examined through two complementary approaches, such as the acetic acid writhing test for visceral pain and the formalin test for inflammatory pain in mice [41]. In the first model, pain is mediated through a localized inflammatory reaction that triggers the liberation of arachidonic acid from membrane phospholipids. This process is facilitated by cyclooxygenase enzymes (both COX‐1 and COX‐2 isoforms), prompted increased biosynthesis of prostaglandins, particularly PGE_2_ and PGF_2_α. Additionally, elevated levels of lipoxygenase‐derived metabolites in peritoneal fluid may contribute to the allogeneic response [42]. Experimental animals writhe in response to acetic acid through a chemosensitive nociceptor. Moreover, the degree of analgesia may be determined by the percentage decrease in stomach squirms [43]. Both doses of APS, at 200 mg/kg and 400 mg/kg, reduced the number of writhes. Thus, the APS exhibited a significant (*p* < 0.01) dose‐dependent analgesic effect, similar to the reference drug diclofenac sodium (*p* < 0.001). Researchers frequently use the formalin test to gain insight into pain and analgesia mechanisms, which provides superior results than studies that use mechanical or heat stimuli [41]. The formalin test evaluates a drug’s efficiency to minimize the extent of time that chronic pain from damaged tissue endures [44]. Formalin generates pain in this test in two periods, each of which connects to a different nociceptive mechanism. Formalin directly affects the nociceptors in the initial phase, and the inflammatory pain response occurs in the late phase. Centrally acting analgesic agents inhibit both phases of the pain response, in contrast to peripherally acting compounds such as diclofenac sodium and corticosteroids, which selectively attenuate only the late phase [41, 45]. In this study, APS exhibited a dose‐dependent antinociceptive effect in the formalin test, with the higher dose (400 mg/kg) producing greater inhibition of paw licking than the lower dose (200 mg/kg). While 200 mg/kg of APS showed limited activity in the early neurogenic phase, 400 mg/kg of APS reduced pain responses more effectively, particularly in the late inflammatory phase. This suggests that the analgesic effect of APS is more pronounced at higher doses and is mainly mediated through suppression of inflammatory pain rather than neurogenic mechanisms.

Carrageenan is widely utilized to estimate the anti‐inflammatory effects of inherent medications since it is known to stimulate tissue macrophages, which in turn generate and release several proinflammatory cytokines, including bradykinin, histamine, tachykinins, TNF‐α, and IL‐1β. Proinflammatory cytokines are known to be produced primarily by activation of several cell‐signaling pathways through reactive oxygen species (ROS) [46]. APS at both 200 and 400 mg/kg doses elicited notable anti‐inflammatory actions, as proven by reduced rat paw edema and increased percent edema protection. After 2 hours, the extracts and the standard medication showed pharmacological activity; this might be explained by the time it takes for the extract and medication to reach the site of action. Hence, the extract is as efficient as diclofenac sodium in reducing rat paw edema and protecting against edema as a percentage. Because COX‐1 and COX‐2 play key roles in prostaglandin production and the mediation of pain and inflammation, both selective and nonselective inhibition of these enzymes can be therapeutically beneficial [47, 48]. Therefore, the compounds identified in APS through GC–MS analysis were subjected to molecular docking against both cyclooxygenase targets. The docking scores ranged from −8.1 to −5.6 kcal/mol for COX‐1 (PDB ID: 5WBE) and −6.7 to −4.4 kcal/mol for COX‐2 (PDB ID: 5IKR). Although the reference drug diclofenac exhibited stronger binding affinity toward COX‐2, several APS constituents demonstrated moderate binding interactions with the cyclooxygenase enzymes. These findings suggest possible interactions with these targets that may contribute to the observed analgesic and anti‐inflammatory activities; however, further experimental studies are required to confirm this potential mechanism.

ADMET analysis is a vital technique for estimating bioactive compounds’ pharmacokinetic and toxicity profiles, along with molecular docking studies [49]. Computational ADMET assessments indicated acceptable safety profiles of the identified phytochemicals from APS. The dug‐likeliness of those phytochemicals was assessed using the Lipinski rule of five, and every compound yielded positive outcomes. While VDss values indicated moderate to high tissue distribution, intestinal absorption was seen above 90% for the majority of the substances, indicating favorable uptake. Some phytoconstituents, including gamma‐sitosterol, phytol, and alpha‐tocopherol, exhibit positive logBBB values, indicating BBB permeability as well as prospective CNS activity.

Despite the promising pharmacological findings, several limitations of the present study should be acknowledged. First, the mechanistic interpretations are based mainly on behavioral models, inflammatory responses, and molecular docking analysis. Therefore, the proposed interactions with COX enzymes and CNS receptors should be considered preliminary and require confirmation through future molecular and biochemical investigations. Second, locomotor activity was not quantitatively assessed using an open‐field or similar test. Although no obvious motor impairment was visually observed during experimentation, behavioral paradigms such as EPM, HBT, FST, and TST may be influenced by sedation or altered motor activity, and thus the specificity of anxiolytic and antidepressant effects should be interpreted cautiously. Third, the study employed five animals per group (*n* = 5), a sample size commonly reported in similar pharmacological research; however, this relatively small number may influence the generalizability of the results. Because behavioral responses in animal models can vary biologically, future studies involving a greater number of animals would help provide stronger validation of the present findings. Finally, while animals were randomly allocated to treatment groups and evaluated using standardized procedures, formal blinding was not implemented during behavioral scoring, which may introduce observer bias. Future studies incorporating blinded assessment, locomotor evaluation, and biochemical assays will be necessary to validate and further elucidate the mechanisms underlying the observed pharmacological effects.

## 5. Conclusion

In summary, the acetone extract of *Pothos scandens* demonstrated significant neuropharmacological, analgesic, and anti‐inflammatory effects in experimental animal models, supporting its traditional medicinal applications. GC–MS analysis identified 11 phytochemical constituents, and in silico docking suggested potential interactions of some compounds with CNS and inflammatory targets. However, these computational findings should be regarded as preliminary indications rather than direct mechanistic confirmation. The present work therefore provides initial pharmacological evidence for the biological potential of *P. scandens*, while further studies involving biochemical‐, molecular‐, and receptor‐level investigations are required to verify the proposed mechanisms and to better evaluate its therapeutic relevance.

## Author Contributions

S. M. Moazzem Hossen: conceptualization, study design, supervision, and writing–review and editing. Jannatul Naima Meem: project administration, investigation, and writing–original draft. Tanjina Nasrin Tamin: investigation, formal analysis, and writing–original draft. Rimon Chowdhury: investigation, formal analysis, and writing–original draft. Sadia Hosna Rony: investigation, formal analysis, and writing–original draft. Sheikh Imrul Kayes: investigation, data curation, and formal analysis. Tawhidul Islam: investigation and writing–review and editing. Md. Liakot Ali: investigation, software, and writing–original draft.

## Funding

This research did not receive any funding.

## Ethics Statement

The study was approved by the Institutional Animal Ethics Review Board, Faculty of Biological Sciences, University of Chittagong, Bangladesh (Approval No: AERB‐FBS‐CU‐2025109). Moreover, the study was conducted in full compliance with internationally recognized guidelines and ethical standards for the humane treatment and use of laboratory animals.

## Conflicts of Interest

The authors declare no conflicts of interest.

## Data Availability

The data that support the findings of this study are available from the corresponding author upon reasonable request.
